# Impaired myoblast differentiation and muscle IGF‐1 receptor signaling pathway activation after N‐glycosylation inhibition

**DOI:** 10.1096/fj.202400213RR

**Published:** 2024-07-04

**Authors:** Giosuè Annibalini, Laura Di Patria, Giacomo Valli, Matteo Bocconcelli, Roberta Saltarelli, Lorenzo Ferri, Laura Barberi, Fabiana Fanelli, Amelia Morrone, Rita Barone, Renzo Guerrini, Antonio Musarò, Vilberto Stocchi, Elena Barbieri

**Affiliations:** ^1^ Department of Biomolecular Sciences University of Urbino Carlo Bo Urbino Italy; ^2^ Department of Clinical and Experimental Sciences University of Brescia Brescia Italy; ^3^ Department of Neuroscience and Medical Genetics Meyer Children’s Hospital IRCCS Florence Italy; ^4^ DAHFMO‐Unit of Histology and Medical Embryology, Laboratory Affiliated to Istituto Pasteur Italia University of Rome La Sapienza Rome Italy; ^5^ Department of NEUROFARBA University of Florence Florence Italy; ^6^ Child Neurology and Psychiatry Unit, Department of Clinical and Experimental Medicine University of Catania Catania Italy; ^7^ Research Unit of Rare Diseases and Neurodevelopmental Disorders Oasi Research Institute‐IRCCS Troina Italy; ^8^ Department of Human Sciences for the Promotion of Quality of Life University San Raffaele Rome Italy

**Keywords:** congenital disorders of glycosylation, glycosylation, IGF1R signaling pathway, muscle atrophy, myoblast differentiation, *PMM2*

## Abstract

The role of N‐glycosylation in the myogenic process remains poorly understood. Here, we evaluated the impact of N‐glycosylation inhibition by Tunicamycin (TUN) or by *phosphomannomutase 2* (*PMM2*) gene knockdown, which encodes an enzyme essential for catalyzing an early step of the N‐glycosylation pathway, on C2C12 myoblast differentiation. The effect of chronic treatment with TUN on tibialis anterior (TA) and extensor digitorum longus (EDL) muscles of WT and MLC/mIgf‐1 transgenic mice, which overexpress muscle *Igf‐1Ea* mRNA isoform, was also investigated. TUN‐treated and *PMM2* knockdown C2C12 cells showed reduced ConA, PHA‐L, and AAL lectin binding and increased ER‐stress‐related gene expression (*Chop* and *Hspa5* mRNAs and *s/uXbp1* ratio) compared to controls. Myogenic markers (*MyoD, myogenin*, and *Mrf4* mRNAs and MF20 protein) and myotube formation were reduced in both TUN‐treated and *PMM2* knockdown C2C12 cells. Body and TA weight of WT and MLC/mIgf‐1 mice were not modified by TUN treatment, while lectin binding slightly decreased in the TA muscle of WT (ConA and AAL) and MLC/mIgf‐1 (ConA) mice. The ER‐stress‐related gene expression did not change in the TA muscle of WT and MLC/mIgf‐1 mice after TUN treatment. TUN treatment decreased *myogenin* mRNA and increased *atrogen‐1* mRNA, particularly in the TA muscle of WT mice. Finally, the IGF‐1 production and IGF1R signaling pathways activation were reduced due to N‐glycosylation inhibition in TA and EDL muscles. Decreased IGF1R expression was found in TUN‐treated C2C12 myoblasts which was associated with lower IGF‐1‐induced IGF1R, AKT, and ERK1/2 phosphorylation compared to CTR cells. Chronic TUN‐challenge models can help to elucidate the molecular mechanisms through which diseases associated with aberrant N‐glycosylation, such as Congenital Disorders of Glycosylation (CDG), affect muscle and other tissue functions.

## Graphical abstract

N‐glycosylation inhibitions by Tunicamycin (TUN) or by knockdown of *phosphomannomutase 2* (*PMM2*) gene block the C2C12 myoblast fusion and impair the myogenic program. TUN treatment decreased myogenic markers and increased atrophy markers in muscles of WT and MLC/mIgf‐1 mice, which overexpress muscle *Igf‐1Ea* mRNA isoform. Finally, the production of N‐glycosylated IGF‐1Ea prohormone and IGF1R signaling pathway activation were markedly inhibited after TUN treatment. Our results offer new insights into impairments of myogenic differentiation capacity in the pathological context of disorders of N‐glycosylation.

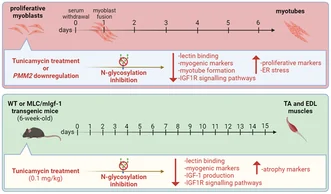

**Abbreviations**
AAL: Aleuria Aurantia (AAL)
AKT: Serine/threonine protein kinase B
CCND1: Cyclin D1
CDG: Congenital Disorders of Glycosylation
Chop: C/EBP homologous protein
Con A: Concanavalin A
CRISPR/Cas9: Clustered regularly interspaced short palindromicrepeats/CRISPR‐associated protein 9
CTR: Control
DAPI: 2‐(4‐amidinophenyl)‐1H‐indole‐6‐carboxamidine
DMEM: Dulbecco’s Modified Eagle Medium
EDL: Extensor digitorum longus muscle
FBS: Fetal bovine serum
GAPDH: Glyceraldehyde‐3‐PhosphateDehydrogenase
Glc‐1,6‐P_2_
: glucose1,6‐bisphosphate
HEK293: Human embryonic kidney 293 cells
HRP: Horseradish peroxidase
Hspa5: Heat Shock Protein FamilyA (Hsp70) Member 5
IGF‐1: Insulin‐like growth factor‐1
IGF1R: IGF‐1 receptor
IR: Insulin Receptor
MF20: Myosin heavy chain
MRF: Myogenic regulatory factors
Mrf4: Myogenic Factor 6
Myf5: Myogenic Factor 5
MyoD: Myogenic differentiation 1
NADP+: oxidized nicotinamide adenine dinucleotide phosphate
NADPH: reduced nicotinamide adenine dinucleotidephosphate
OST: Oligosaccharyltransferase
PBS: Phosphate buffered saline
PCNA: Proliferating Cell NuclearAntigen
PHA‐L: Phaseolus vulgaris leucoagglutinin
PI3K: Phosphatidylinositol 3 kinase
PMM2: Phosphomannomutase 2
SA‐βgal: Senescence‐associated beta‐galactosidase
TA: Tibialis anterior muscle
TUN: Tunicamycin
WT: wild type
Xbp1: spliced X‐box binding protein 1

## 1 INTRODUCTION

Myogenesis is a complex and highly regulated process that requires myoblast proliferation, alignment of cells, and subsequent fusion into multinucleated myotubes.^1^ Many steps of myogenesis can be recapitulated *in vitro* through myogenic cell lines such as mouse C2C12 and rat L6E9, which in the absence of mitogenic stimuli form multinucleated myotubes.^1^ The myogenic process depends on a complex network of signal transduction pathways regulating the expression of myogenic regulatory factors (MRFs), a group of basic helix–loop–helix transcription factors that include MyoD, Myf5, myogenin, and Mrf4.^1^, ^2^ Besides the canonical MRF, other molecules such as insulin‐like growth factor‐1 (IGF‐1) are directly involved in the myogenesis process.^3^ The primary effects of IGF‐1 are mediated by binding to the IGF‐1 receptor (IGF1R), a ligand‐activated receptor tyrosine kinase. IGF1R signal transduction occurs through the PI3K/AKT/mTOR and RAF/MEK/ERK pathways which promote muscle protein synthesis and inhibit muscle protein degradation.^4^ Recent studies showed that both IGF‐1 and IGF1R are synthetized as pre‐protein precursors which are extensively processed to allow IGF‐1 secretion and the formation of functional IGF1R, respectively.^5^, ^6^, ^7^, ^8^, ^9^ In particular, IGF‐1 is synthesized as a larger prohormone that is subsequently converted into mature IGF‐1 by furin cleavage of its C‐terminal extension named E‐peptide. Due to alternative splicing of the *Igf‐1* gene, three different E‐peptides may be produced: Ea‐, Eb‐, and Ec‐peptides (named according to human nomenclature). Ea‐peptide contains two N‐glycosylation sites in mice,^9^, ^10^ one of which is also conserved in humans (N92).^5^ We demonstrated that N‐glycosylation of the human IGF‐1Ea prohormone is necessary for efficient IGF‐1 secretion.^5^ On the other hand, both the IGF‐1Eb and IGF‐1Ec prohormones lacked putative N‐glycosylation sites, and their production appears to be largely unaffected by glycosylation.^5^ Proper N‐glycosylation is essential also for IGF1R translocation to the plasma membrane and for IGF‐1‐induced activation.^6^, ^7^, ^8^, ^11^ Thus, the IGF‐1 signaling pathway seems to be particularly sensitive toward N‐glycosylation.^12^ Growing evidence highlights the role of protein glycosylation in myoblast fusion and differentiation.^13^, ^14^, ^15^, ^16^, ^17^ Gundry R.L. et al. identified 128 glycosylated proteins in the C2C12 myoblast cell surface, including Insulin Receptor (IR) and IGF1R.^18^ More recently, Blazev R. et al. demonstrated a dynamic change in proteomic, N‐glycomic, and N‐glycoproteomic during rat L6 myotube formation.^17^ Interestingly, the abundance of lectins and enzymes involved in glycan biosynthesis and remodeling were modulated after the differentiation of myoblasts toward myotubes, suggesting that temporal‐ and site‐specific glycosylation tuning is important for skeletal muscle cell development, differentiation, and function.^17^ During myoblast differentiation, increased binding of lectins with different carbohydrate specificities^19^, ^20^ and regulation of N‐glycan content of glycoproteins,^17^ including several integrins and growth factor receptors, has been shown.

The effects of incorrect protein muscle glycosylation in humans are illustrated by dystroglycanopathies, a group of inherited genetic diseases caused by defective O‐mannosylation of ɑ‐Dystroglycan.^21^ However, the consequence of aberrant protein N‐glycosylation in muscle homeostasis remains poorly understood. Early evidence showed that fusion of embryonic quail muscle cells is impaired by Tunicamycin (TUN), an antibiotic that inhibits the Dolichyl‐Phosphate N‐Acetylglucosaminephosphotransferase 1 (DPAGT1) enzyme which catalyzes the first step of the N‐glycosylation pathway.^22^ More recently, Xia et al. demonstrated that administration of TUN increases ER‐stress markers in the liver, decreases hepatic *Igf‐1* mRNA expression and IGF‐1 serum levels, and reduces body growth and body weight gain in C57BL/6J mice.^23^ However, the impact of TUN on muscle tissues is still unknown.

A wide spectrum of neuromuscular syndromes ranging from muscle dystrophy to congenital myopathy/myasthenic syndrome has been associated with Congenital Disorders of Glycosylation (CDG) caused by N‐glycosylation defects.^24^ CDGs are inborn errors of metabolism following a Mendelian pattern of inheritance characterized by “substantial hypoglycosylation in one or more cell types”.^25^ Over 160 CDG types and more than 200 phenotypes have been characterized.^25^ The most common type is PMM2‐CDG, caused by pathogenetic variants in the *PMM2* gene encoding the enzyme phosphomannomutase 2 (PMM2) that converts mannose‐6‐phosphate into mannose‐1‐phosphate, the obligatory precursor for GDP‐mannose production and hence N‐linked glycosylation.^26^ The global suppression of N‐glycosylation in PMM2‐CDG affects multiple systems leading to a variety of symptoms. Patients with PMM2‐CDG exhibit prominent cerebellar ataxia due to progressive cerebellar atrophy in addition to other neuromuscular symptoms such as peripheral neuropathy which leads to causes functional disability and wheelchair dependence.^27^, ^28^ The rate and the severity of myopathy in PMM2‐CDG have not been extensively explored.

In the present study, we evaluate the consequences of N‐glycosylation inhibition on myoblast differentiation by TUN administration or *PMM2* knockdown in mouse C2C12 model cells. Moreover, 6‐week‐old wild‐type (WT) and MLC/mIgf‐1 transgenic FVB mice, which overexpress the *Igf‐1Ea* isoform under the control of the myosin light chain (MLC) promoter,^29^ were chronically challenged with a sub‐phenotypic dose of TUN to mimic a mild CDG‐like phenotype. Tibialis anterior (TA) and extensor digitorum longus (EDL) muscles were collected to evaluate the impact of N‐glycosylation inhibition on lectin binding, ER‐stress‐related gene expression, and IGF‐1 signaling pathway activation.

## 2 MATERIALS AND METHODS

### 2.1 Cell cultures

C2C12 mouse myoblasts (Sigma‐Aldrich, Italy), CRISPR‐CTR and CRISPR‐PMM2 C2C12, and rat L6E9 myoblasts^30^ were cultured in Dulbecco's Modified Eagle Medium (DMEM) supplemented with 10% heat‐inactivated fetal bovine serum (FBS), 2 mM glutamine, penicillin (100 U/mL), and streptomycin (100 μg/mL), and maintained in a 5% CO_2_ atmosphere at 37°C. To induce C2C12 myogenic differentiation, C2C12 myoblasts at 80%–90% confluence were transferred to a differentiation medium (DM) containing 2% horse serum, as previously described.^31^ To activate myogenic differentiation of rat L6E9, myoblasts were switched to a DM containing DMEM plus 1% bovine serum albumin.^30^ The C2C12 was maintained in the DM for 6 days, while L6E9 for 4 days. For TUN (cat. no. T7765; Sigma‐Aldrich) treatment, C2C12 and L6E9 myoblasts were incubated with 0.01 μg/mL of TUN for 24 h and then for 6 days (C2C12) or 4 days (L6E9) in the DM. The medium was changed every 2 days along with fresh DMSO (control) or TUN treatment. Cells were harvested on day 1 (C2C12 and L6E9) and days 4 (L6E9) and 6 (C2C12) after the induction of the differentiation. C2C12 myoblasts concentration and viability were determined by LUNA‐II™ Automated Cell Counter (Logos Biosystems, Twin Helix) with trypan blue staining. Cytotoxicity was calculated as follows: Cytotoxicity (%) = [(dead cell number/total cells) × 100]. All cell lines were routinely screened for the absence of mycoplasm contamination by PCR using the Mycoplasm Detection kit (abm, cat. num G238, SIAL srl, Italy). All mutant cell lines are available from the authors upon request.

### 2.2 WT and MLC/mIgf1 transgenic mice

Jackson Laboratories' FVB female mice were utilized as embryo donors for the generation of MLC/mIgf1 transgenic animals, as previously described.^29^ Animals were kept in ventilated cages (4–5 per cage) in a room with constant temperature (22°C) and humidity (45%–55%), and with 12:12 h light/dark cycle. Each cage was equipped with wood shavings, bedding and a cardboard tube for enrichment and spontaneous physical activities. The mice had access to a constant supply of food (Teklad Global 18% Protein Rodent Diet; Envigo, Huntingdon, UK) and water. Every four months, sentinel mice were examined for any potential infection listed in the FELASA recommendations. Female WT and MLC/mIgf1 transgenic animals of 6 weeks of age were used for all the experiments. Animals were randomly allocated to sham or TUN groups. The research project was conducted according to the animal welfare regulations and guidelines and approved by the Institutional Review Board of the animal facilities of the National Institute of Health—Italy.

### 2.3 PMM2 knockdown by CRISPR/Cas9

For CRISPR/Cas9 silencing, 5.0 × 10^4^ C2C12 cells/well were seeded in 12‐well plates and transfected with 1 μg of the PMM2 Double‐Nickase plasmid (cat. no. sc‐424790‐NIC; Santa Cruz Biotechnology, D.B.A. Italia) or the corresponding Double‐Nickase Control plasmid (cat. no. sc‐437281; Santa Cruz Biotechnology, D.B.A. Italia) using the TransIT‐X2® Transfection Reagent (Mirus Bio, TEMA ricerca) for 48 h. Following the manufacturer's protocol, the selection was performed with puromycin (cat. no. P9620; Sigma‐Aldrich), and clones were screened by qRT‐PCR and PMM enzymatic activity assay. Three C2C12 myoblast clones with a marked reduction of *PMM2* mRNA expression and PMM enzymatic activity were selected for further analyses.

### 2.4 PMM enzymatic activity assay

CRISPR‐CTR and CRISPR‐PMM2 C2C12 cells were washed twice in PBS, scraped, pelleted by centrifugation, and resuspended in lysis buffer (20 mM Hepes, 25 mM KCl, 1 mM dithiothreitol, and 1× protease inhibitor mixture) and then briefly sonicated and incubated at 4°C for 15 min. Afterward, the cell lysates were centrifuged at 15 000 rpm for 10 min at 4°C to remove cellular debris. Protein concentration of each freshly prepared cell lysate was determined with Bradford Reagent (Sigma‐Aldrich). PMM enzymatic activity was assayed as reported by Van Schaftingen and Jaeken^32^ with slight modification. Briefly, PMM activity was measured at 32°C in Hepes 20 mM pH 7.5 containing MgCl_2_ 5 mM, NADP^+^ 0.25 mM, in the presence of 0.3 mM Mannose‐1‐P as the substrate, 1 μM Glc‐1,6‐P_2_ as the activator, 2.8 U/mL glucose 6‐phosphate dehydrogenase, 3.7 U/mL phosphoglucose isomerase, and 3.9 μg/mL phosphomannose isomerase. The reaction started by adding 50 μg/mL of cell lysates. The activity was followed spectrophotometrically for 60 min at 340 nm, recording the reduction of NADP^+^ to NADPH. One unit is the amount of enzyme that catalyzes the conversion of 1 μmol of substrate per min under this condition.

### 2.5 Immunofluorescence

C2C12 and L6E9 cell cultures were washed with phosphate‐buffered saline (PBS) (8 g/L NaCl, 1.15 g/L Na_2_HPO_4_, 0.2 g/L KH_2_PO_4_, 0.2 g/L KCl), fixed for 15 min at room temperature with 4% formaldehyde/PBS and permeabilized in 0.5% Triton X‐100/PBS for 4 min. A mouse monoclonal antibody against Myosin heavy chain (MF20 at the 1:2 dilution; obtained from DSHB) was incubated at 37°C for 1 h followed by incubation at 37°C for a 30‐min with 1:100 fluorescein‐conjugated goat anti‐mouse IgG (Biolegend, San Diego, CA, USA). An antibody against total IGF1R β (1:1000; cat. n. 3027 Cell Signaling Technology) was incubated at room temperature for 1 h followed by incubation at room temperature for 1 h with 1:200 Rhodamine Red™‐X (RRX) AffiniPure™ Goat Anti‐Rabbit IgG (H+L) (Jackson ImmunoResearch Europe Ltd.). Cells were stained with DAPI (2‐(4‐amidinophenyl)‐1H‐indole‐6‐carboxamidine) for nuclear visualization and mounted in Mowiol 4‐88 (Sigma‐Aldrich) slides. Images were acquired using the TOUPCAM™ E31SPM05000KPA digital camera connected to a Leica microscope DMLB and the ToupView software. Fusion index was determined by counting the number of nuclei in differentiated myotubes using ImageJ software and expressed as a percentage of the total number of nuclei.

### 2.6 RNA extraction, cDNA synthesis, and qRT‐PCR

Total RNA was extracted and purified using the Omega Bio‐Tek E.Z.N.A.™ Total RNA kit (VWR International) according to the manufacturer's instructions. The amount of RNA was assessed with SpectraMax® QuickDrop™ Micro‐Volume Spectrophotometer (Molecular Devices, CaRli biotec), and the complementary DNA was synthesized from 500 ng of total RNA using Takara PrimeScript™RT Master Mix (Takara Bio Inc., Diatech Lab Line Srl). Subsequently, quantitative RT‐PCR (qRT‐PCR) was performed with 2 μL of cDNA and 300 nM of each primer in an Applied Biosystems StepOnePlus™ Real‐Time PCR System using PowerUp SYBR Green Master Mix (Applied Biosystems). The qRT‐PCR conditions were 50°C for 2 min, 95°C for 2 min followed by 40 cycles of two steps at 95°C for 15 s, and 60°C for 60 s. The relative mRNA expression of target genes was normalized to *gapdh* internal control. The genes of interest and the sequence of the specific primer used in qRT‐PCR quantification are listed in Table S1.

### 2.7 Immunoblotting and lectin blotting

Cells were lysed adding 40–60 μL of lysis buffer containing: 20 mM HEPES (pH 7.9), 25% v/v glycerol, 0.42 M NaCl, 0.2 mM EDTA, 1.5 mM MgCl_2_, 0.5% v/v Nonidet P‐40, 1 mM DTT, 1 mM NaF, 1 mM Na_3_VO_4_, and 1× complete protease inhibitor cocktail (Roche Diagnostics). The lysates were frozen and thawed twice and clarified by centrifugation at 12 000 rpm for 10 min at 4°C. Protein concentration in each sample was determined using the Bradford colorimetric assay (Bio‐Rad Laboratories) and the DU‐640 UV Spectrophotometer (Beckman Coulter). The protein samples (20–30 μg total proteins) were electrophoresed through 10% SDS‐PAGE, and then transferred to nitrocellulose or PVDF membranes (Bio‐Rad Laboratories) for immunoblotting. Primary antibodies against phospho‐IGF1R β (1:2000; cat. n. 3024 Cell Signaling Technology), total IGF1R β (1:2000; cat. n. 3027 Cell Signaling Technology), phospho‐Akt (Ser473) (1:2000; cat. n. 9271 Cell Signaling Technology), total Akt (1:2000; cat. n. 9272 Cell Signaling Technology), phospho‐p44/42 (ERK1/2) (1:2000; cat. n. 9101 Cell Signaling Technology), total p44/42 (ERK1/2) (1:2000; cat. n. 9102 Cell Signaling Technology), PCNA (1:5000, cat. n. MAB 424R Millipore), MF20 (1:500, DSHB), and mouse/rat biotinylated IGF‐1 antibody (1:300, cat. n. BAF791 R&D Systems) were incubated overnight at 4°C. For lectin blotting, membranes were probed with biotinylated Concanavalin A (Con A, 1:1000), Phaseolus vulgaris leucoagglutinin (PHA‐L, 1:200), and Aleuria Aurantia (AAL, 1:400) lectins (Vector laboratories, D.B.A. Italia) at room temperature while shaking for 1 h. After washes, the membranes were incubated with the appropriate horseradish peroxidase (HRP)‐conjugated secondary antibody (against primary antibodies) or streptavidin‐HRP (for IGF‐1 and biotinylated lectins) at room temperature for 1 h and were then washed three times. Deglycosylation of TA and EDL muscle proteins was performed by incubation tissue lysates with 2.5 U of PNGase F (Sigma‐Aldrich; cat. num. P7367) for 2 h at 37°C, according to the manufacturer's recommendations. An aliquot of tissue lysates incubated with an equal volume of PNGase assay reaction buffer without the enzyme PNGase F was used as a control. Blots were developed using Clarity Western ECL Substrate (Bio‐Rad Laboratories) and were quantified using the ChemiDoc MP (Bio‐Rad Laboratories) equipped with Image Lab software.

### 2.8 CRISPR‐CTR and CRISPR‐PMM2 cellular senescence quantification

Cellular senescence was evaluated via detection of the SA‐βgal activity in CRISPR‐CTR and CRISPR‐PMM2 cells. Wild‐type cells were also treated with 600 μM hydrogen peroxide (H_2_O_2_) for 2 h and served as a positive control.^33^ Myoblasts at 80%–90% confluence were washed twice with PBS, fixed for 5 min at room temperature with 4% paraformaldehyde, and washed again three times in PBS. Cells were then incubated overnight at 37°C without CO_2_ with freshly prepared staining solution at pH 6 according to Chen et al.^33^ After the incubation, six random images per well (10× objective) were collected using a digital camera (ColorView, Soft Imaging Systems) adapted to an inverted microscope (Olympus CKX41). The senescent cells responding to the SA‐βgal activity (identified by a blue ring usually around the nucleus) were manually counted with the ImageJ software, and the ratio of senescent cells was calculated.

### 2.9 Statistical analysis

Sample size for cell culture experiments was determined based on practicability and prior experience with the C2C12^31^ and L6E9^30^ cells. The numbers of mice to be used were derived from a power analysis conducted using preliminary data for lectin binding (mean ± SD) in WT mice treated with TUN and considering 80% power an alpha level of significance of .05. Data are represented as mean ± SD of at least three independent experiments. The biological replicate numbers are indicated as the n numbers in the figure legends. Variables of interest were checked for normal distribution using the Shapiro–Wilks test, and parametric statistical analysis was employed to determine significant differences. Results for mRNA expression of *myogenin*, *Mrf4*, *Chop*, *Hspa5*, and *s/uXBP1* ratio were not normally distributed and therefore log‐transformed and reanalyzed. Statistical analyses were performed using Student's *t*‐test or two‐way ANOVA as appropriate, followed by Tukey's multiple comparison post hoc tests. A *p*‐value <.05 was considered statistically significant.

## 3 RESULTS

### 3.1 TUN treatment and C2C12 myoblast differentiation

To mimic a mild CDG‐like phenotype, we first aimed to find the minimal dose of TUN for C2C12 myoblasts that would interfere with glycosylation while maintaining cell viability. The optimal TUN treatment of C2C12 myoblasts was established to be 0.01 μg/mL since cells retained the capacity to exclude trypan blue (cytotoxicity CTR = 1.7% ± 1.8%; TUN 0.01 μg/mL for 72 h = 1.8% ± 0.5; *p* = .92) for up to 72 h. At the highest concentration (0.05 μg/mL), cytotoxic effects became apparent, and the myoblasts appeared highly vacuolated and started to detach from the dish surfaces (cytotoxicity CTR = 1.7% ± 1.8%; TUN 0.05 μg/mL for 72 h = 50.5% ± 3.3%; *p* < .0001). Thus, a concentration of 0.01 μg/mL was used to evaluate the effect of chronic low‐dose TUN treatment on C2C12. TUN was added to proliferating C2C12 myoblasts 24 h before and during 6 days of cell differentiation. Cells were harvested on days 1 and 6 after the induction of differentiation. First, we used Con A, PHA‐L, and AAL lectins to analyze the glycosylation pattern of C2C12 (Figures 1A and S1A). Con A recognizes oligomannose and hybrid glycans mannose, PHA‐L recognizes complex‐type N‐glycans, while AAL binds to fucose linked (α ‐1,6) to N‐acetylglucosamine or to fucose linked (α ‐1,3) to N‐acetyllactosamine. As shown in Figure 1A, we found a significant increase in Con A, PHA‐L, and AAL reactivity at day 6 compared to day 1, indicating a rise in high mannose, complex N‐glycans, and fucosylated proteins, respectively. Notably, the lectin binding profile of TUN‐treated cells was similar to CTR at day 1 but markedly differed at day 6, showing a general reduction of N‐glycosylation. The expression level of ER‐stress‐related genes *Chop* and *Hspa5* and the *s/uXBP1* ratio increased after TUN treatment at both days 1 and 6 compared to CTR, as expected (Figure 1B). Subsequently, we analyze the effect of TUN treatment on C2C12 differentiation by immunofluorescence analysis of the myosin heavy chain (MF20) (Figure 1C). CTR cells appeared as long multinucleated cells at day 6 (fusion index of 53.8 ± 8.0%), while myotube formation was markedly inhibited in TUN‐treated cells (fusion index at day 6 = 18.2 ± 10.7%). We then examined the mRNA levels of myogenic markers *Ccnd1*, *MyoD*, *myogenin*, and *Mrf4* during differentiation (Figure 1D). In CTR cells, *Ccnd1* was expressed on day 1 and was downregulated on day 6, as expected. *MyoD* and *myogenin* showed the opposite trend. In TUN‐treated cells, *Ccnd1* was not downregulated on day 6 but appeared to increase. In addition, TUN‐treated cells expressed significantly lower levels of *MyoD*, *myogenin*, and *Mrf4* mRNAs compared to CTR on days 1 and 6. Finally, we found that TUN treatment increased the PCNA protein expression on day 1 and markedly reduced the MF20 protein expression compared to CTR on day 6 (Figure 1E,F). We found that TUN treatment inhibited the differentiation (Figure S2A), increased *Ccnd1* mRNA expression on day 1, and decreased *MyoD*, *myogenin*, and *Mrf4* mRNA levels on day 4 (Figure S2B) of rat L6E9 myoblasts, similar to C2C12 myoblasts, which indicate that N‐glycosylation has a crucial role in myoblast differentiation.

**FIGURE 1:**
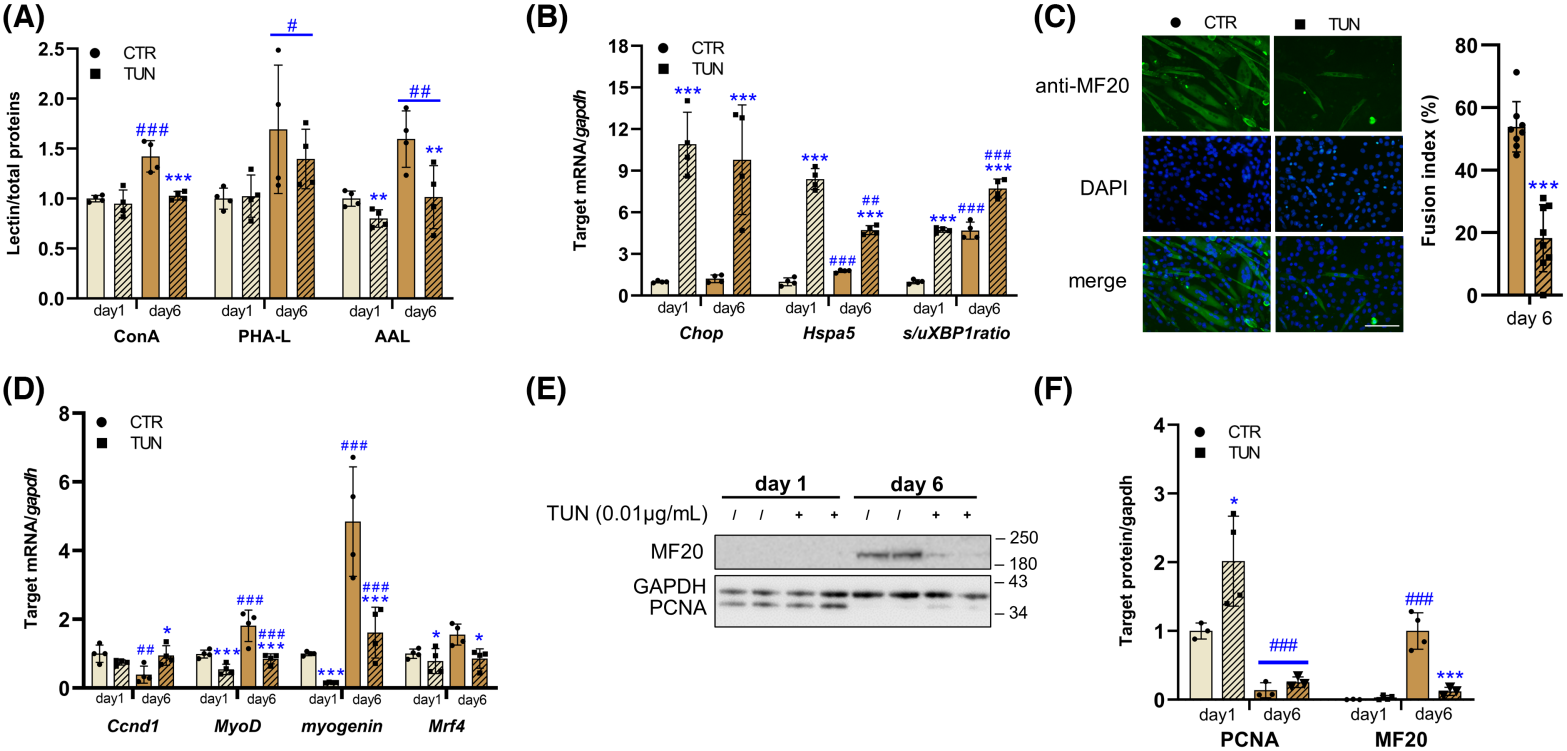
Effect of TUN treatment on C2C12 cell differentiation. (A) Quantification of lectin binding in C2C12 treated with TUN (0.01 μg/mL); C2C12 cells were collected on days 1 and 6 (*n* = 4). AAL, *Aleuria aurantia* lectin; ConA, concanavalin A; PHA‐L, *Phaseolus vulgaris* leucoagglutinin. Representative lectin blots were presented in Figure S1A. (B) Expression of ER‐stress‐related genes *Chop*, *Hspa5*, and *s/uXBP1* ratio in C2C12 cells treated with TUN (0.01 μg/mL) (*n* = 4). (C) Effects of TUN treatment (0.01 μg/mL) on myotubes formation at day 6 (*n* = 8), the scale bar represents 100 μm. (D) Quantification of *Ccnd1*, *MyoD*, *myogenin*, and *Mrf4* mRNA expression by qRT‐PCR (*n* = 4). (E) Representative immunoblotting and (F) band densitometry analysis of C2C12 treated with 0.01 μg/mL of TUN using antibodies directed against PCNA and MF20. All bar charts are presented as mean values ± SD. Significant differences were determined using unpaired *t*‐test (C) or two‐way ANOVA followed by Tukey's multiple comparison post hoc tests (A, B, D, F). *Significantly different compared to CTR; ^#^Significantly different compared to day 1; * and ^#^ *p* ≤ .05; ** and ^##^ *p* ≤ .01, *** and ^###^ *p* ≤ .001.

### 3.2 PMM2 knockdown and C2C12 myoblast differentiation

Subsequently, we knocked down *PMM2* in C2C12 by stable transfection of *PMM2* CRISPR/Cas9 double‐nickase plasmid to analyze the effects of PMM2 deficiency on myoblast differentiation. RNA from PMM2‐targeted cells (CRISPR‐PMM2) and control plasmid‐transfected cells (CRISPR‐CTR) was collected to confirm the effective targeting of the *PMM2* gene. We obtained three myoblast clones with a marked reduction in *PMM2* mRNA expression compared with the corresponding CRISPR‐CTR clones (10.7 ± 2.8 fold reduction at day 1; *p* < .0001). Accordingly, CRISPR‐PMM2 myoblasts had a significantly reduced PMM enzyme activity (CRISPR‐CTR at day 1 = 1.64 ± 0.4 mU/mg of proteins; CRISPR‐PMM2 at day 1 = 0.34 ± 0.25 mU/mg of proteins; *p* < .01) and the downregulation of *PMM2* mRNA expression also persisted in cells harvested at day 6 after differentiation (16.9 ± 4.0 fold reduction; *p* < .0001). The lectin binding profile of CRISPR‐PMM2 cells showed a reduction of lectins binding at day 6 compared to CRISPR‐CTR (Figures 2A and S1B). The analyses of the expression level of ER‐stress‐related genes in CRISPR‐PMM2 cells showed a slight increase of *s/uXBP1* ratio on day 1 and a marked increase of *Chop* and *Hspa5* mRNA level and *s/uXBP1* ratio on day 6 compared to CRISPR‐CTR (Figure 2B). We next examined the differentiation potential of CRISPR‐PMM2 myoblasts. As shown in Figure 2C, CRISPR‐PMM2 cells failed to form myotubes at day 6, showing a marked reduction in the myogenic index (CRISPR‐CTR fusion index at day 6 = 51.0 ± 8.7%; CRISPR‐PMM2 fusion index at day 6 = 0.9 ± 0.6%) (Figure 2C). We found an increase of *Ccnd1* mRNA and decrease *MyoD* and *myogenin* mRNA levels in CRISPR‐PMM2 at day 1 compared to CRISPR‐CTR cells (Figure 2D), while the PCNA protein expression did not change (Figure 2E,F). Conversely, *MyoD*, *myogenin*, and *Mrf4* mRNA levels (Figure 2D) and MF20 protein expression (Figure 2E,F) strongly decreased at day 6. The analysis of senescence‐associated beta‐galactosidase (SA‐βgal) activity showed no difference between CRISPR‐PMM2 and CRISPR‐CTR myoblast (% senescence CRISPR‐CTR = 2.5 ± 2.4; CRISPR‐PMM2 = 2.2 ± 2.1; *p* = .85), suggesting that the lack of differentiation cannot be attributed to senescence in the selected CRISPR‐PMM2 clones.

**FIGURE 2:**
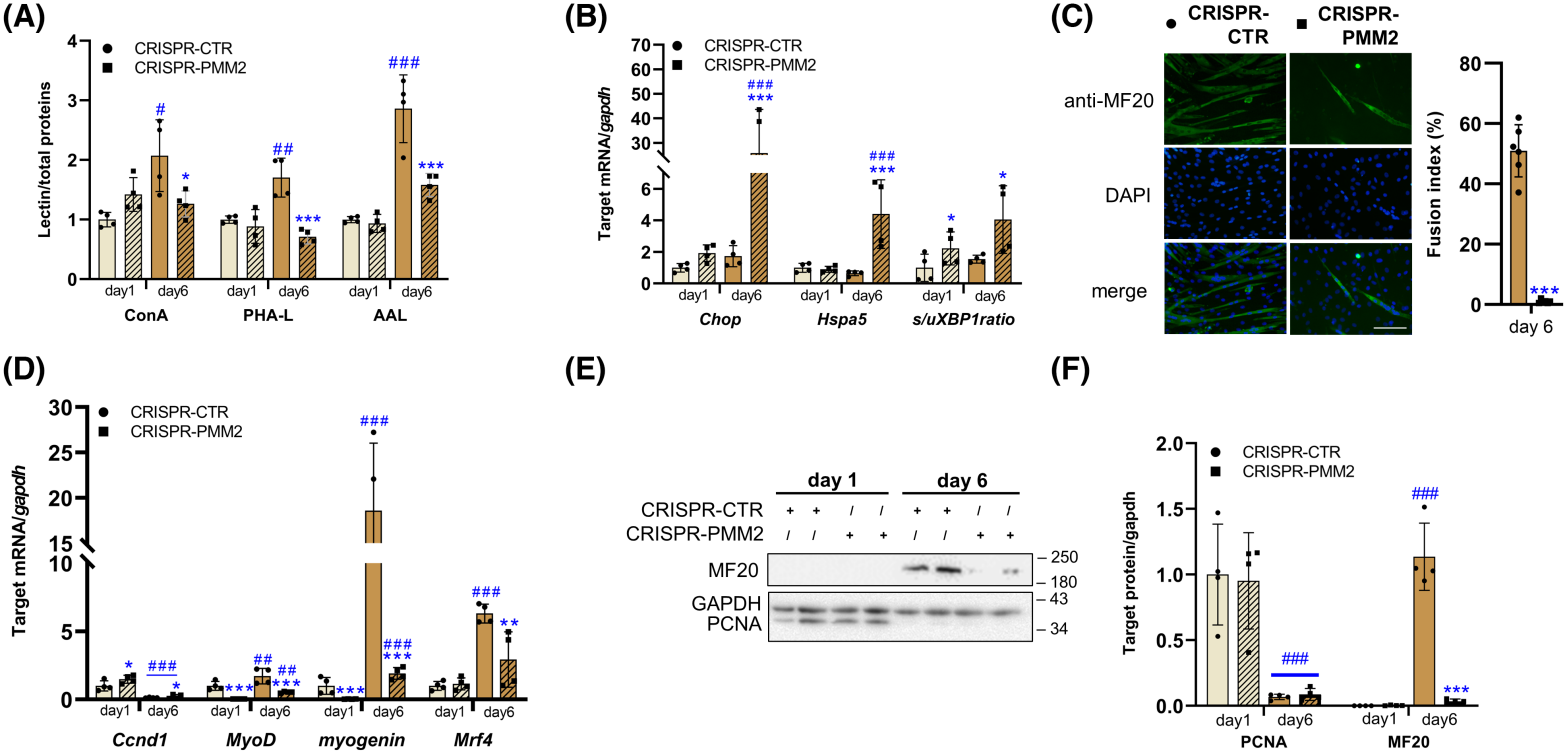
Effect of PMM2 downregulation on C2C12 differentiation. (A) Quantification of lectin binding after knockdown of *PMM2* gene by CRISPR/Cas9 (CRISPR‐PMM2) and control plasmid‐transfected cells (CRISPR‐CTR). AAL, *Aleuria aurantia* lectin; ConA, concanavalin A; PHA‐L, *Phaseolus vulgaris* leucoagglutinin. Representative lectin blots were presented in Figure S1B. (B) Expression of ER‐stress‐related genes *Chop*, *Hspa5*, and *s/uXBP1* ratio in CRISPR‐CTR and CRISPR‐PMM2 C2C12; C2C12 cells were collected on days 1 and 6 (*n* = 4). (C) Effects of PMM2 knockdown on myotubes formation at day 6 (*n* = 6), the scale bar represents 100 μm. (D) Quantification of *Ccnd1*, *MyoD*, *myogenin*, and *Mrf4* mRNA expression by qRT‐PCR (*n* = 4). (E) Representative immunoblotting and (F) band densitometry analysis of CRISPR‐CTR and CRISPR‐PMM2 C2C12 using antibodies directed against PCNA and MF20. All bar charts are presented as mean values ± SD. Significant differences were determined using unpaired *t*‐test (C) or two‐way ANOVA followed by Tukey's multiple comparison post hoc tests (A, B, D, F). *Significantly different compared to CTR; ^#^Significantly different compared to day 1; * and ^#^ *p* ≤ .05; ** and ^##^ *p* ≤ .01, *** and ^###^ *p* ≤ .001.

### 3.3 Effects of chronic treatment with low TUN dose on TA muscle lectin binding, ER‐stress‐related gene expression, and muscle markers of myogenesis and atrophy

To investigate the role of N‐glycosylation inhibition on muscle *in vivo*, 6‐week‐old WT and MLC/mIgf‐1 mice were chronically challenged with a low TUN dose (0.1 mg/kg for 15 days). MLC/mIgf‐1 mice showed increased body and TA muscle weight compared to WT mice (Figure 3A), as expected.^29^ Conversely, body and TA muscle weight were similar between CTR and TUN‐treated mice (Figure 3A). TUN treatment decreased ConA lectin binding in the TA muscle of WT and MLC/mIgf‐1 mice, while AAL reactivity markedly decreased only in TA muscle of WT mice (Figures 3B and S1C). The ER‐stress‐related gene expression did not change in the TA muscle of WT and MLC/mIgf‐1 mice (Figure 3C). In TA muscle of WT and MLC/mIgf‐1 mice, the mRNA expression of *myogenin* decreased after TUN treatment, while *atrogin‐1* expression levels increased only in the WT mice (Figure 3D).

**FIGURE 3:**
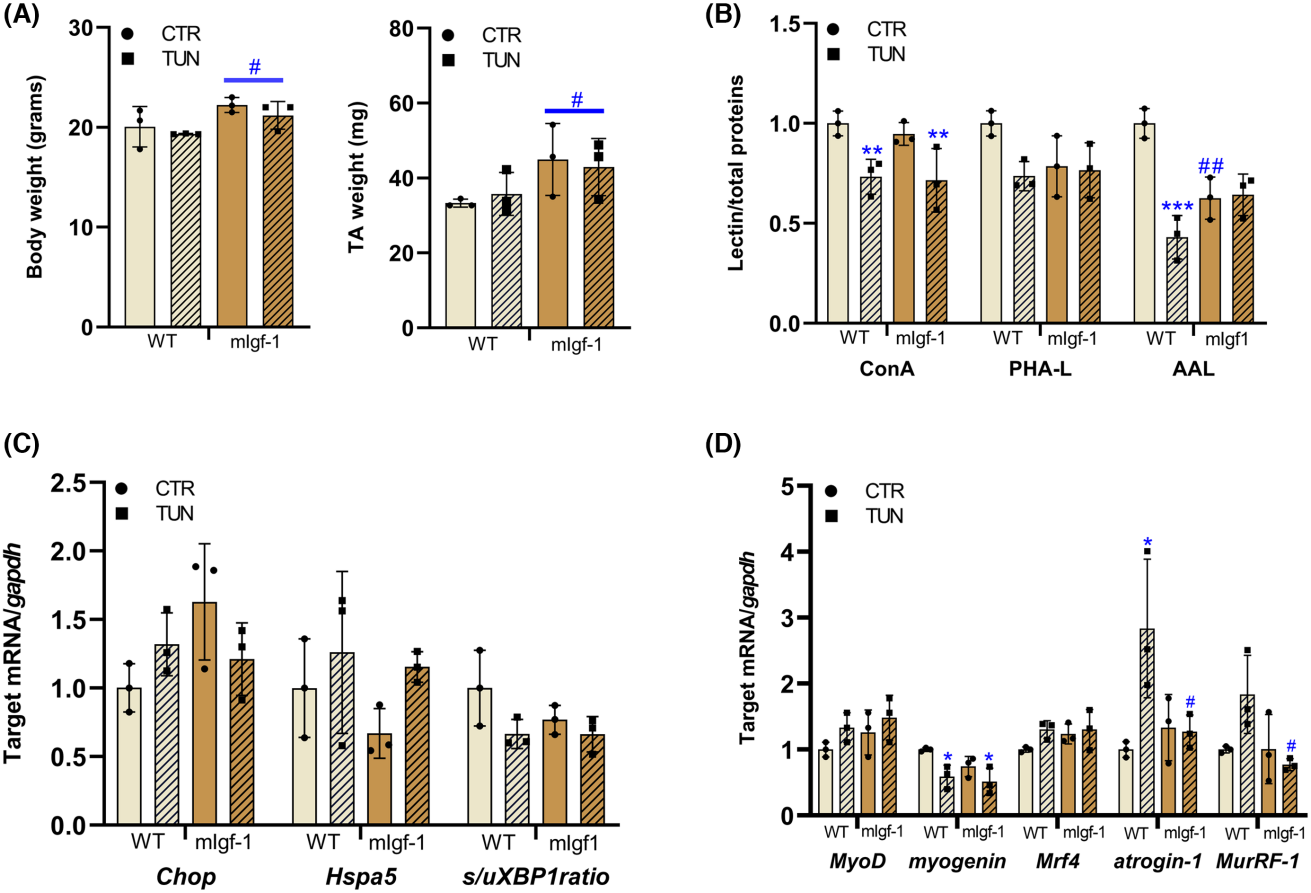
Effect of chronic treatment with low TUN dose (0.1 mg/kg of TUN for 15 days) on TA muscle of WT and MLC/mIgf‐1 mice. (A) Body and TA muscle weight of WT (*n* = 3) and MLC/mIgf‐1 (*n* = 3) mice treated with TUN for 15 days. (B) Quantification of lectin binding in the TA muscle of WT (*n* = 3) and MLC/mIgf‐1 (*n* = 3) mice treated with TUN; AAL, *Aleuria aurantia* lectin; ConA, concanavalin A; PHA‐L, *Phaseolus vulgaris* leucoagglutinin. Representative lectin blots were presented in Figure S1C. (C) Expression of ER‐stress‐related genes *Chop* and *Hspa5* and *s/uXBP1* ratio in the TA muscle of WT (*n* = 3) and MLC/mIgf‐1 (*n* = 3) mice treated with TUN. (D) Quantification of *MyoD*, *myogenin*, *Mrf4, atrogin‐1*, and *MuRF‐1* mRNA expression by qRT‐PCR in the TA muscle of WT (*n* = 3) and MLC/mIgf‐1 (*n* = 3) mice treated with TUN (*n* = 3). All bar charts are presented as mean values ± SD. Significant differences were determined using two‐way ANOVA followed by Tukey's multiple comparison post hoc tests. *Significantly different compared to CTR; ^#^Significantly different compared to WT mice; * and ^#^ *p* ≤ .05; ** and ^##^ *p* ≤ .01, ***p ≤ .001.

### 3.4 Effects of chronic treatment with low TUN dose on *Igf‐1* mRNA and IGF‐1 protein expression and on IGF1R pathways activation in the TA muscle of WT and MLC/mIgf1 mice and in C2C12 myoblasts

The expression of endogenous *Igf‐1Ea* and *Igf‐1Ec* isoforms slightly decreased after TUN treatment in TA (Figure 4A) and EDL (Figure S3A) muscle of WT and MLC/mIgf1 mice, although this reduction was statistically significant only in TA muscle. We also measured the effect of TUN treatment on the expression of *mIgf‐1* transgene by quantifying total *Igf‐1* (*Igf‐1tot*) mRNA. It is important to note that the primer set used to amplify the expression of *mIgf‐1* transgene (i.e., *Igf‐1 tot*) also recognizes the endogenous *Igf‐1* mRNA isoforms (i.e., *Igf‐1Ea* and *Igf‐1Ec* mRNAs). However, the *mIgf‐1* transgene is highly expressed compared to the endogenous *Igf‐1* gene (2^−ΔΔCT^ = 59.5 ± 10.5), and hence the *Igf‐1Ea* and *Igf‐1Ec* mRNA quantity was negligible compared to that of *mIgf‐1* transgene. Interestingly, we found that the *mIgf‐1* transgene was unaffected by the TUN treatment in the TA (Figure 4A) and EDL (Figure S3A) muscles of MLC/mIgf1 mice. Western blot analyses showed that the TA (Figure 4B) and EDL (Figure S3B) muscles of untreated MLC/mIgf1 mice mainly expressed IGF‐1Ea prohormones with three immunoreactive bands of ~22, ~17, and ~12 kDa. The ~22 and ~17 kDa bands disappeared after the deglycosylation with PNGase F, resulting in an accumulation of the ~12 kDa band (Figure S4). Thus, the ~22 and ~17 kDa bands likely represent two N‐glycosylated forms of the IGF‐1Ea prohormone, while the lower band of ~12 kDa represents the unglycosylated IGF‐1Ea prohormone. No IGF‐1 immunoreactive signals were detected in the TA and EDL muscles of WT mice, probably because the amount of IGF‐1 was below the detection limit of Western blot. Band densitometry analysis showed that TUN treatment decreased the 22 and 12 kDa bands in the TA muscle (Figure 4C) and those of 17 and 12 kDa in the EDL muscle (Figure S3C) of MLC/mIgf‐1 mice. Finally, TA muscle of MLC/mIgf1 mice showed increased IGF1R phosphorylation compared to WT mice, which was markedly inhibited by TUN treatment (Figure 4D,E). Accordingly, AKT and MAPK ERK1/2 phosphorylation in the TA muscle of WT and MLC/mIgf1 mice decreased after TUN treatment. Similar results were obtained in the EDL muscle of WT and MLC/mIgf1 mice (Figures S3D,E). The effect of TUN treatment on IGF1R signaling pathway was also investigated on C2C12 harvested on day 1 (Figure 5). Interestingly, we found a marked reduction of total IGF1R and increased mobility shift of IGF1R proreceptor in TUN‐treated cells (Figure 5A), suggesting that TUN treatment altered the IGF1R proreceptor processing and total IGF1R quantity, as previously described.^8^, ^11^ Subsequently, we analyzed the sub‐cellular localization of IGF1R by immunofluorescence staining (Figure 5B). C2C12 CTR cells showed mainly perinuclear and diffuse cytoplasmic pattern, which was markedly reduced after TUN treatment. Altogether, these results showed that N‐glycosylation inhibition affects the IGF1R signaling pathway activation due to the reduction of IGF‐1 production and IGF1R activity. To evaluate if IGF1R reduction observed after TUN treatment was associated with reduced IGF1R signaling pathway activation, C2C12 were treated with recombinant mouse IGF‐1 (100 ng/mL) for 30 min, and the level of IGF1R, AKT, and MAPK ERK1/2 phosphorylation was quantified by Western blotting (Figure 5C,D). This analysis demonstrated that the IGF‐1‐induced activation of IGF1R signaling pathway was markedly reduced in TUN‐treated C2C12 compared to CTR cells.

**FIGURE 4:**
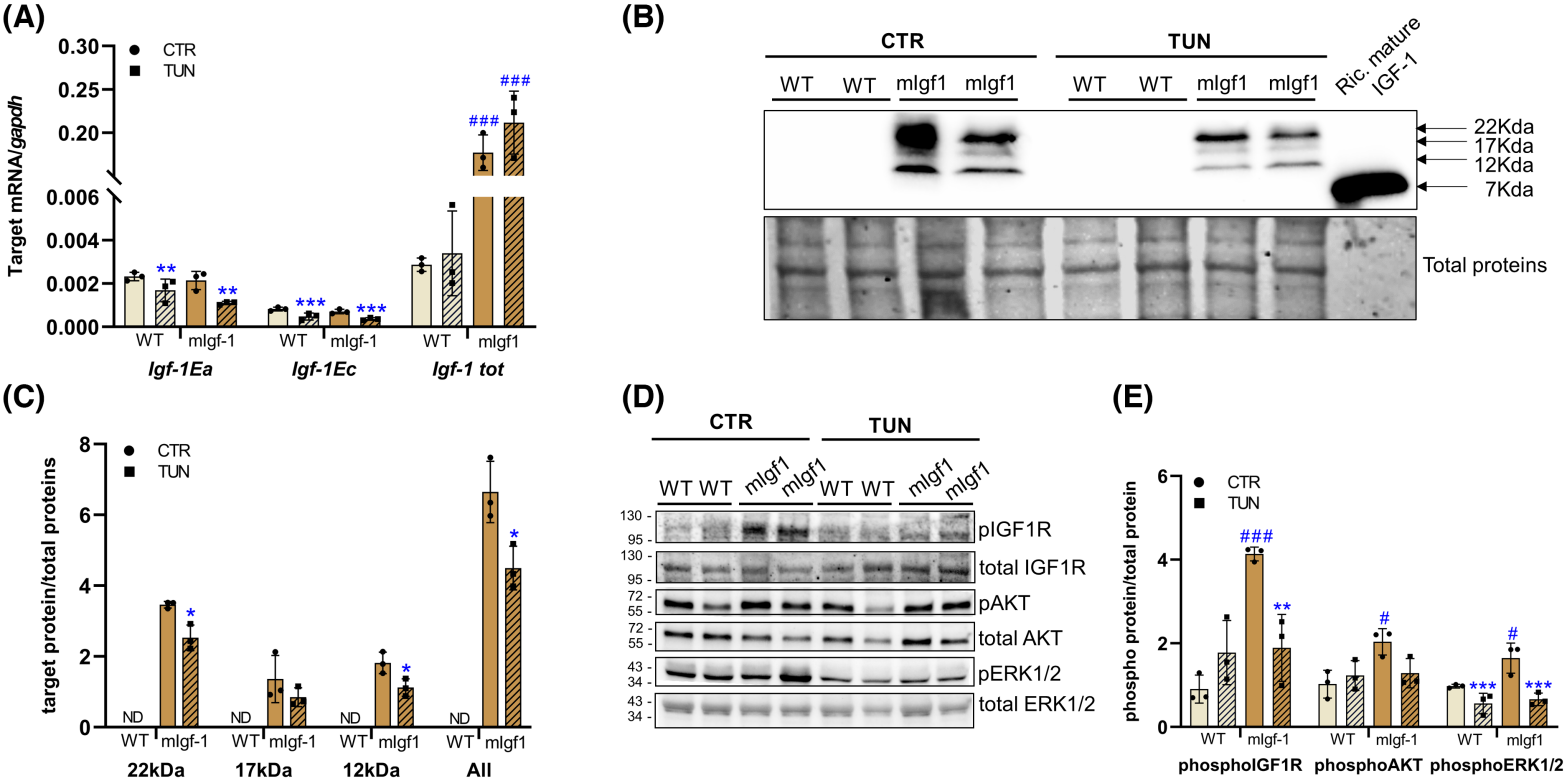
IGF‐1 production and IGF1R signaling pathway activation in TA muscle of WT and MLC/mIgf‐1 mice treatment with low TUN dose (0.1 mg/kg of TUN for 15 days). (A) Expression of endogenous *Igf‐1* mRNA isoforms (i.e., *Igf‐1Ea* and *Igf‐1Ec* mRNAs) and total *Igf‐1* (i.e., endogenous and mIgf‐1 transgene mRNAs) in the TA muscle of WT (*n* = 3) and MLC/mIgf‐1 (*n* = 3) mice treated with TUN. (B) Representative immunoblotting and (C) band densitometry analysis of TA muscle of WT and MLC/mIgf1 mice using an antibody directed against mature mouse IGF‐1 sequence. Three bands at a molecular weight of ~22, ~17, and ~12 kDa were detected in TA muscle of MLC/mIgf1 mice. Recombinant mouse IGF‐1 mature protein was loaded as a positive control for mature IGF‐1 (~7 kDa). (D) Representative immunoblotting and (E) band densitometry analysis of TA muscle of WT and MLC/mIgf1 mice using antibodies directed against phosphorylated (pIGF1R) and total IGF1R, phosphorylated (pAKT) and total AKT, and phosphorylated (pERK1/2) and total ERK1/2. All bar charts are presented as mean values ± SD. Significant differences were determined using unpaired *t*‐test (C) or two‐way ANOVA followed by Tukey's multiple comparison post hoc tests (A, E). *Significantly different compared to CTR; ^#^Significantly different compared to WT mice; * and ^#^ *p* ≤ .05; ** *p* ≤ .01 *** and ^###^ *p* ≤ .001.

**FIGURE 5:**
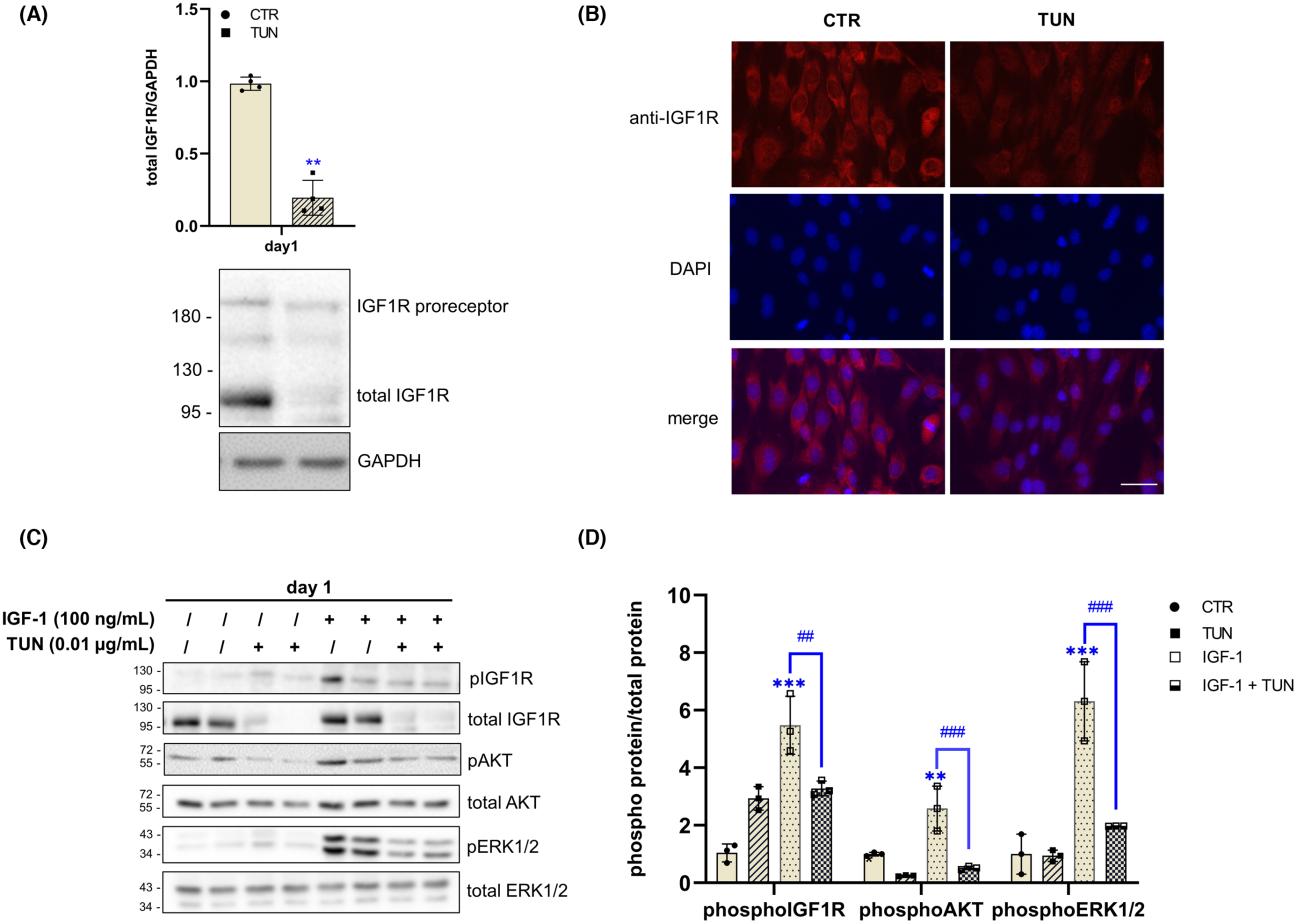
Effect of TUN treatment on IGF1R production and IGF1R signaling pathway activation in C2C12. (A) IGF1R and IGF1R proreceptor production in C2C12 cells treated with 0.01 μg/mL of TUN and harvested at day 1. (B) Immunofluorescence analysis of C2C12 cells stained with ant‐IGF1R and DAPI, the scale bar represents 200 μm. (C) Representative immunoblotting and (D) band densitometry analysis of IGF‐1‐induced IGF‐1R signaling pathway activation in CTR‐ and TUN‐treated C2C12 cells using antibodies directed against phosphorylated (pIGF1R) and total IGF1R, phosphorylated (pAKT) and total AKT, and phosphorylated (pERK1/2) and total ERK1/2. The calculation of pIGF1R level was normalized against total lysed protein instead of total IGF1R to account for the marked reduction of total IGF1R after TUN treatment. All bar charts are presented as mean values ± SD. Significant differences were determined using unpaired *t*‐test (A) or one‐way ANOVA followed by Tukey's multiple comparison post hoc tests (D). *Significantly different compared to CTR, ^#^Significantly different compared to IGF‐1‐treated cells; ** *p* ≤ .01, *** *p* ≤ .001; ^##^ *p* ≤ .01, ^###^ *p* ≤ .001.

## 4 DISCUSSION

In this study, we used two *in vitro* strategies to study the effects of N‐glycosylation inhibition on C2C12 myoblast differentiation, one based on TUN treatment and one based on PMM2 downregulation. Chronic treatment with a low dose of TUN, intended to prompt a mild impairment of N‐glycosylation resembling a mild CDG phenotype, was also administrated in WT and MLC/mIgf‐1 mice in order to investigate the effects of N‐glycosylation inhibition on muscle *in vivo*. TUN is an inhibitor of DPAGT1 at the first step in the N‐glycosylation pathway and prompts a deficiency of N‐linked glycans.^34^ TUN is also an ER‐stress activator due to the accumulation of hypoglycosylated and misfolded proteins.^35^ In untreated C2C12 cells, we observed an increased binding of ConA, PHA‐L, and AAL lectins during myoblast differentiation. This finding is in keeping with other studies and suggests that muscle cell glycosylation changes during differentiation.^19^, ^20^ A comparison of differentiated and undifferentiated C2C12 cells shows that glycoproteins derived from myotubes bear higher oligomannose/hybrid‐type N‐glycan (ConA), complex‐type N‐glycan (PHA‐L), and fucosylated (AAL) residues. Interestingly, C2C12 cells differentiated under TUN treatment exhibited a lectin binding pattern more similar to the undifferentiated myoblasts. We found an increase in ER‐stress‐related gene expression (*Chop* and *Hspa5* mRNAs and *s/uXbp1* ratio) in C2C12 myoblast and myotubes treated with TUN compared to those untreated, as expected. Interestingly, TUN‐induced hypoglycosylation decreased myogenic markers and prevented myotubes formation in C2C12. Previous studies have shown that doses from 1.0 to 10 μg/mL of TUN are toxic in C2C12 cells, induce strong ER‐stress pathways activation, inhibit mitosis, and increase apoptosis.^36^ However, the effects of high TUN dose on myoblast differentiation are still unclear, probably due to its toxicity and off‐target effects.^22^, ^36^, ^37^ Here, we found that low TUN doses (i.e., concentration ≤ 0.01 μg/mL) do not have toxic effects on C2C12 myoblasts and preserve myoblast proliferation (we observed increased PCNA protein levels). Hence, the inhibition of C2C12 differentiation induced by non‐toxic doses of TUN is likely to be caused by the TUN‐induced hypoglycosylation instead of unexpected secondary effects (i.e., mitosis arrest and apoptosis) described in other studies.^22^, ^36^ To confirm the detrimental effect of hypoglycosylation on myoblast differentiation, we produced C2C12 cells with stable downregulation of *PMM2*. The PMM2 protein is an enzyme that converts mannose‐6‐phosphate into mannose‐1‐phosphate, the immediate precursor of GDP‐mannose which is a key substrate for the synthesis of glycans. PMM2 is the most frequently mutated enzyme in human CDG. Living PMM2‐CDG patients harbor hypomorphic alleles and residual PMM2 activity because the complete loss of PMM2 function is lethal.^38^ We found that the PMM2 downregulation decreased lectin binding in myotubes while the ER‐stress‐related gene expression increased, as expected. Interestingly, the differentiation process and myotube formation were markedly inhibited after PMM2 dowregulation, corroborating results obtained in TUN‐treated cells. Nevertheless, CRISPR cells also showed some small differences with respect to C2C12 challenged with TUN and parental WT cell population. In particular, *Chop* and *Hspa5* mRNA levels markedly increased at day 1 and still remained high at day 6 after TUN treatment, whereas these ER‐stress markers increased only at day 6 in CRISPR‐PMM2 cells. Moreover, the magnitude of relative expression changes of *Chop*, *myogenin*, and *MRF4* mRNAs was higher in CRISPR compared to parental WT cells. A slightly different phenotype between CRISPR C2C12 clonal derivatives and parental cells was expected,^39^ although we verified that CRISPR‐CTR clones maintain differentiation capability. Moreover, both CRISPR‐CTR and CRISPR‐PMM2 myoblasts did not show cellular senescence as demonstrated by β‐galactosidase assay. An alternative possibility to explain the difference between TUN‐treated and CRISPR‐PMM2 cells might be related to the disease mechanism behind each model since mutations in *DPAGT1* gene, which encodes the target of TUN, caused the DPAGT1‐CDG which is characterized by a more severe clinical phenotypes compared to PMM2‐CDG.^40^, ^41^ Thus, although both TUN‐treated and CRISPR‐PMM2 cells showed reduced lectin binding and myoblast differentiation, future efforts should be made to create models that fully mimics the PMM2 mutations found in patients and to investigate the impact of PMM2 deficiency on myogenic differentiation using primary patient‐derived myoblasts. PMM2‐CDG is associated with a broad range of clinical symptoms including generalized hypotonia, distal muscle atrophy, and wasting also related to peripheral neuropathy. Signs of myopathy can be moderate (6% of patients) to severe (2% of patients) in the natural history of PMM2‐CDG.^42^ Recently, Muthusamy K et al. demonstrated that about 82% of the 51 patients included in the studied PMM2‐CDG population exhibited clinical myopathy, suggesting that myopathy is a commonly overlooked aspect of this disease.^43^ Although uncommon in infancy, cardiomyopathy can also occur in PMM2‐CDG and represents a life‐threatening condition.^44^, ^45^ Observation of muscle histology is very rarely described in PMM2‐CDG, but mild myopathic changes regarding fiber size variation and myofibrillar disarray have been reported.^46^ Mildly elevated serum creatine kinase can also occur.^47^ Muscle‐specific *PMM2* knockdown causes qualitative movement defects in *Drosophila*.^48^ Although the pathophysiology of hypotonia in PMM2‐CDG is probably complex, our results suggest that the hypoglycosylation‐induced defective differentiation of myoblasts into myotubes could be a contributing factor.

Xia et al. recently demonstrated that a chronic administration of TUN to C57BL/6 mice induces growth retardation (reduced weight, height, and tibia length) in association with low IGF‐1 production and signaling.^23^ Moreover, it has been shown that N‐glycosylation has an essential function in protein folding, stability, and intracellular trafficking of both IGF‐1 prohormone and IGF1R proreceptor.^5^, ^6^, ^49^ Low IGF1R expression in primary fibroblasts derived from CDG patients with deficient N‐glycosylation^49^, ^50^ and in HEK293 cells lacking the catalytically active subunit of the Oligosaccharyltransferase (OST)^6^ has been shown. Furthermore, desialylation of IGF1R decreased the proliferative response of L6 myoblasts^51^ and receptor activity in a cell model of GNE myopathy.^52^ We therefore evaluated the IGF‐1 signaling pathway activation in TA and EDL muscles of WT and MLC/mIgf‐1 transgenic mice treated with chronic (15 days) low dose of TUN. We found that TUN reduced ConA lectin reactivity in TA muscle from WT and MLC/mIgf‐1 mice and AAL binding in MLC/mIgf‐1 mice. ER‐stress‐related gene expression did not change in the TA muscle of WT and MLC/mIgf‐1 mice. Since continuous low‐dose administration of TUN has a relatively mild impact on glycosylation and ER‐stress marker expression, we can suppose that this condition resembles a mild CDG phenotype. The myogenic marker *myogenin* was downregulated in the TA muscles of WT and MLC/mIgf‐1 mice after low‐dose TUN treatment. Moreover, the mRNA expression of muscle atrophy marker *atrogin‐1* was increased in WT mice after TUN treatment. Interestingly, we found a slight reduction of endogenous *Igf‐1Ea and Igf‐1Ec* mRNA isoforms in association with TUN administration in TA muscle of both WT and MLC/mIgf‐1 mice; however, this decrease did not reach statistical significance in EDL muscle. Moreover, *mIgf‐1* transgene overexpression in MLC/mIgf‐1 mice did not change after TUN treatment. Thanks to the overexpression of *Igf‐1Ea* in the MLC/mIgf‐1 mice, we were also able to analyze the impact of TUN on muscle IGF‐1Ea proteins by Western blot. Muscle IGF‐1 was mainly expressed as proIGF‐1Ea not mature IGF‐1, as previously shown.^5^, ^10^ The expression level of proIGF‐1Ea, including the ~22 kDa band, likely representing an N‐glycosylated form of proIGF‐1Ea,^5^, ^10^, ^49^ decreased after TUN treatment. IGF1R and AKT activation were reduced after TUN treatment in the muscle of MLC/mIgf‐1 mice while both WT and MLC/mIgf‐1 mice showed reduced MAPK ERK1/2 phosphorylation compared to untreated mice. The reduction of IGF‐1 prohormone production and IGF1R signaling pathway activation was also observed in EDL muscle of WT and MLC/mIgf‐1 mice treated with TUN. Moreover, using C2C12 myoblasts, we demonstrated reduced IGF1R expression and increased IGF1R proreceptor mobility shift after TUN treatment, which was associated with marked reduction of IGF1R, AKT, and MAPK ERK1/2 activation following exogenous IGF‐1 treatment. Given the important role played by the IGF1R signaling pathway on myoblast proliferation and muscle cell growth,^3^, ^53^ our results suggested that reduced IGF1 production and IGF1R signaling activation may be involved in the impaired muscle development and function found in CDG. However, the molecular effectors underlying the pathophysiology of muscle involvement in CDG are only starting to be unraveled, and further studies are needed to clarify the involved biological pathways. In this regard, chronic TUN‐challenge mouse models can help elucidate the molecular mechanisms through which diseases associated with aberrant N‐glycosylation, differentially affect tissue, and organ function.

## AUTHOR CONTRIBUTIONS

Giosuè Annibalini and Elena Barbieri: Conceptualization, Formal analysis, Funding acquisition, Resources, Data curation, Writing – Reviewing and Editing; Laura Di Patria, Giacomo Valli, Matteo Bocconcelli, and Fabiana Fanelli: Investigation, Visualization; Roberta Saltarelli, Lorenzo Ferri, and Laura Barberi Investigation, Visualization, Writing – Original draft preparation; Amelia Morrone, Rita Barone, Renzo Guerrini, Antonio Musarò, and Vilberto Stocchi: Supervision, Funding acquisition, Resources, Writing – Reviewing and editing. All the authors contributed to manuscript revision, and read and approved the submitted version.

## DISCLOSURES

None of the authors have any conflicts of interest or financial ties to disclose.

## Supporting information


**Figure S1.**.

## Data Availability

The data that support the findings of this study are available in the methods and/or supplementary material of this article.
