# Supplementary material for: Impaired myoblast differentiation and muscle IGF‐1 receptor signaling pathway activation after N‐glycosylation inhibition
Source: FASEB J. 2024 Jul 4;38(13):e23797. doi: 10.1096/fj.202400213RR (PMC13688064; doi:10.1096/fj.202400213RR)
Supplement: Supplementary file 1 [file FSB2-38-e23797-s001.pdf]

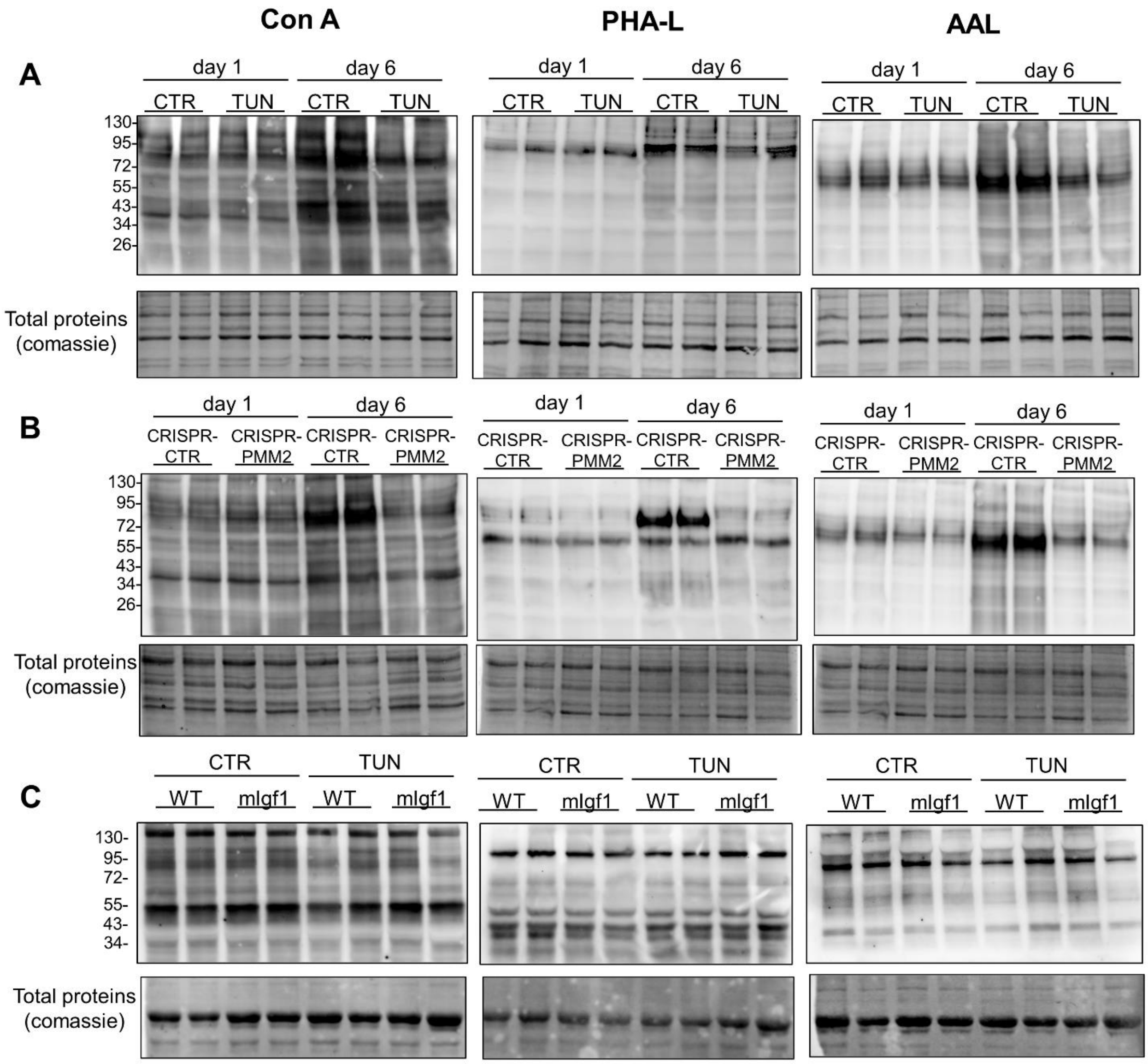

Figure S1: (A) Representative blots of Con A, PHA-L and AAL lectin binding after TUN treatment of C2C12 cells; (B) CRISPR-CTR and CRISPR-PMM2 C2C12 cells: and (C) Tibialis anterior (TA) muscle of CTR and TUN-treated WT and MLC/mIgf1 mice. Biological replicates of C2C12 cells (n=2), WT (n=2) and MLC/mIgf1 mice (n=2) were shown. TUN, tunicamycin; ConA, Concanavalin A; PHA-L, *Phaseolus vulgaris* leucoagglutinin; AAL, *Aleuria aurantia* Lectin.

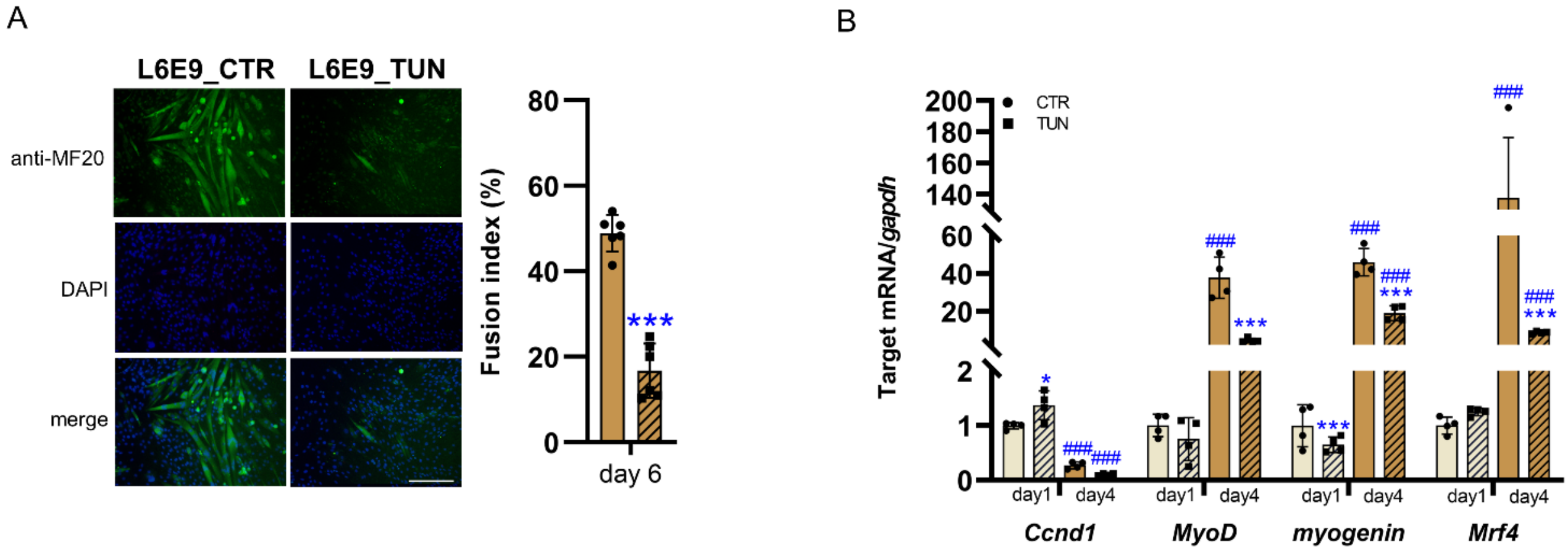

Figure S2. Effect of TUN treatment on rat L6E9 cell differentiation. (A) Effects of TUN treatment (0.01 µg/ml) on L6E9 myotubes formation at day 4 (n=6), the scale bar represents 100 µm. (B) Quantification of *Ccnd1*, *MyoD*, *myogenin* and *Mrf4* mRNA expression by qRT-PCR; cells were collected on days 1 and 4 (n=4). All bar charts are presented as mean values ± s.d. Significant differences were determined using unpaired T-test (A) or two-way ANOVA followed by Tukey's multiple comparison post hoc tests (B). *Significantly different compared to CTR; #Significantly different compared to day 1; * $p \leq 0.05$; *** and ### $p \leq 0.001$.

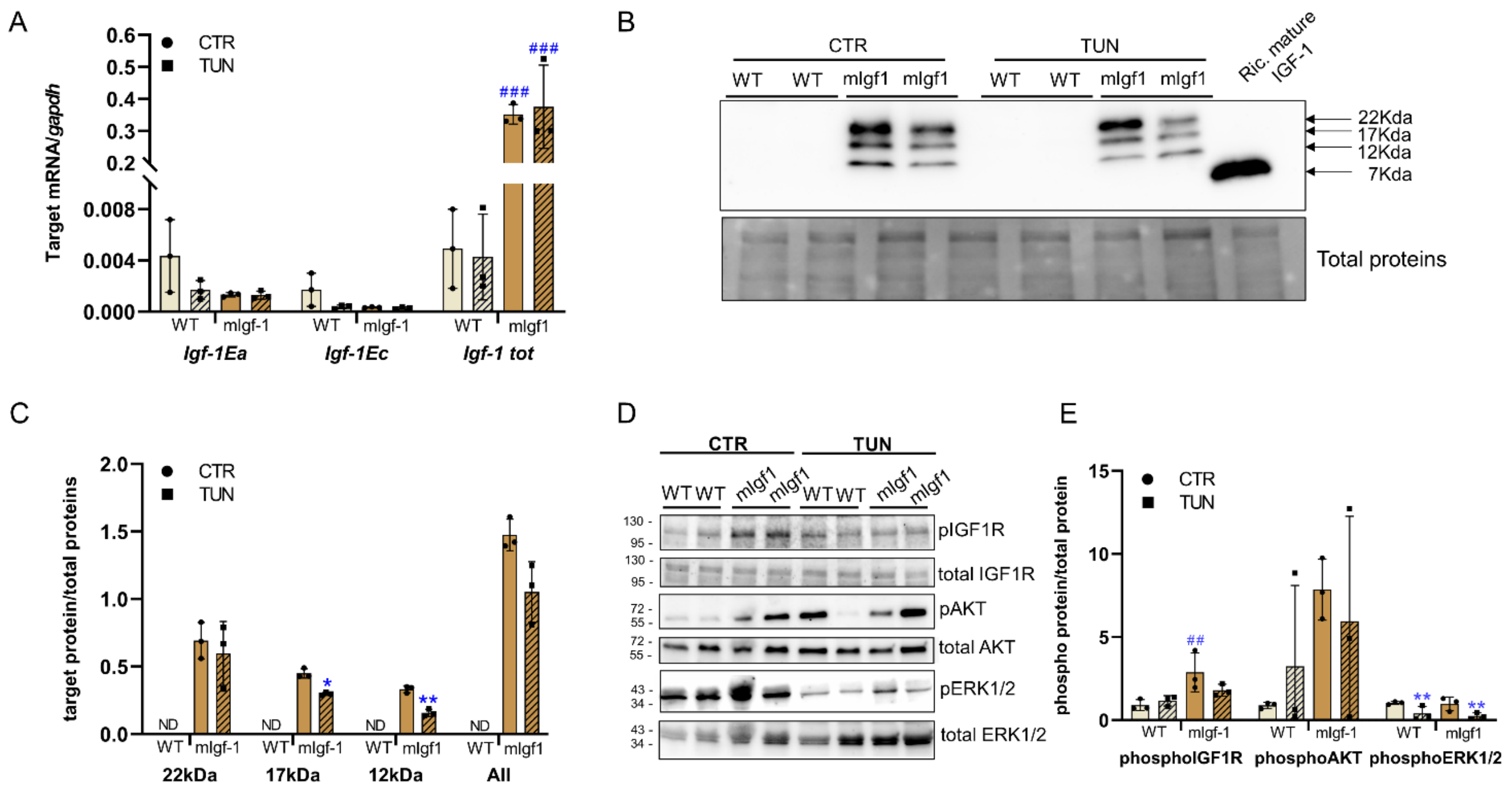

Figure S3. IGF-1 production and IGF1R signalling pathway activation in EDL muscle of WT and MLC/mIgf-1 mice treatment with low TUN dose (0.1 mg/kg of TUN for 15 days). (A) Expression of endogenous *Igf-1* mRNA isoforms (i.e. *Igf-1Ea* and *Igf-1Ec* mRNAs) and total *Igf-1* (i.e. endogenous and exogenous *Igf-1* mRNAs) in the EDL muscle of WT (n = 3) and MLC/mIgf-1 (n = 3) mice treated with TUN. (B) Representative immunoblotting and (C) band densitometry analysis of EDL muscle of WT and MLC/mIgf1 mice using an antibody directed against mature mouse IGF-1 sequence. Three bands at a molecular weight of ~22, ~17 and ~12 kDa were detected in EDL muscle of MLC/mIgf1 mice. Recombinant mouse IGF-1 mature protein was loaded as a positive control for mature IGF-1 (~7 kDa). (D) Representative immunoblotting and (E) band densitometry analysis of EDL muscle of WT and MLC/mIgf1 mice using antibodies directed against phosphorylated (pIGF1R) and total IGF-1R, phosphorylated (pAKT) and total AKT, and phosphorylated (pERK1/2) and total ERK1/2. All bar charts are presented as mean values ± s.d. Significant differences were determined using unpaired T-test (C) or two-way ANOVA followed by Tukey's multiple comparison post hoc tests (A, E). *Significantly different compared to CTR; #Significantly different compared to WT mice; ** and ## p ≤ 0.01.

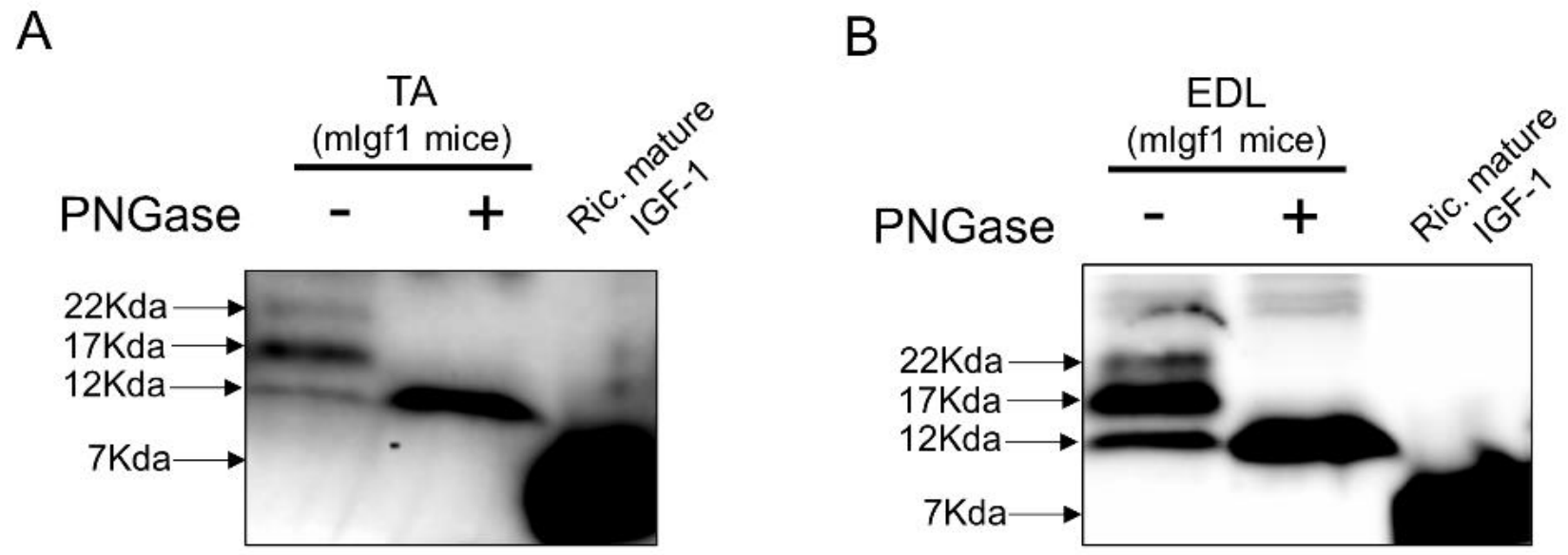

Figure S4: Deglycosylation of (A) Tibialis anterior (TA) and (B) EDL proteins using the *N*-Glycosidase F (PNGase F). The ~22 and ~17 kDa bands disappeared after PNGase treatment determining an accumulation of the band at ~12 kDa, which probably corresponds to unglycosylated IGF-1Ea prohormone.

Table S1. Primer sequences used in qRT-PCR quantification.

Gene	Species	Primer forward (5'-3')	Primer reverse (5'-3')	Purpose
Ccnd1	mouse and rat	CTTCCTCTCCAAAATGCCAG	TGGAGGGTGGGTTGGAAAT	Quantification of the expression level
MyoD	mouse	TTCTTCACCACTCCTCTGAC	GCCGTGAGAGTCGTCTTAT	Quantification of the expression level
MyoD	rat	TTTTTCATGCGACTCACAGC	CTGTGGGAAAGAGTGGGTGT	Quantification of the expression level
Myogenin	mouse	CAACCCAGGAGATCATTG	CATATCCTCCACCGTGATGC	Quantification of the expression level
Myogenin	rat	ACTCCCTTACGTCCATCGTG	CAGGACAGCCCCACTTAAAA	Quantification of the expression level
Mrf4	mouse	GTGGCCAAGTGTTTCGGAT	AAAGGCGCTGAAGACTGC	Quantification of the expression level
Mrf4	rat	GGCCAAGTGTTTCGGATCAT	TCCTGCTGAGCGAAGAATGT	Quantification of the expression level
PMM2	mouse	TTGCCTGAGACACCTGGAAC	AGATCCTGCGTGTGTCTTCG	*PMM2* knockdown confirmation
*Igf-1Ea**	mouse	GCCCAAGACTCAGAAGGAAGTAC	CGGTGATGTGGCATTTTCTG	Endogenous mRNA quantification
*Igf-1Ec/mgf**	mouse	GCTGCAAAGGAGAAGGAAAG	Same as *Igf-1Ea* reverse primer	Endogenous mRNA quantification
Igf-1 tot[#]	mouse and rat	GCTATGGCTCCAGCATTCG	TCCGGAAGCAACACTCATCC	Transgene mRNA expression
Gapdh	mouse and rat	GGCAAATTCAACGGCACAGT	CGCTCCTGGAAGATGGTGAT	Endogenous reference

Note: *Ccnd1*, cyclin D1; *MyoD*, myogenic differentiation 1; *MRF4*, myogenic factor 6; *Myf5*, myogenic factor 5; *PMM2*, phosphomannomutase 2; *Igf-1*, insulin-like growth factor-1 isoform; *GAPDH*, gliceraldeyde-3-phosphate dehydrogenase.* The *Igf1Ea* and *Igf1Ec/mgf* primer sets respectively quantified the endogenous *Igf-1Ea* and *Igf-1Ec/mgf* mRNA level while the *mIgf-1* transgene was not amplified with these primer sets since the reverse primer was designed outside the *mIgf-1* cDNA sequence used in the *mIgf-1* transgene. [#] The *Igf-1 tot* primer set amplified both the endogenous *Igf1-Ea* and *Igf1-Ec/mgf* mRNAs and the *mIgf-1* transgene, however since the expression of endogenous *Igf-1* isoforms was negligible compared to that of *mIgf-1* transgene the *Igf-1 total* primer set actually quantified the transgene *mIgf-1* mRNA expression.
